# Calcium Supplementation Improves Na^+^/K^+^ Ratio, Antioxidant Defense and Glyoxalase Systems in Salt-Stressed Rice Seedlings

**DOI:** 10.3389/fpls.2016.00609

**Published:** 2016-05-12

**Authors:** Anisur Rahman, Kamrun Nahar, Mirza Hasanuzzaman, Masayuki Fujita

**Affiliations:** ^1^Laboratory of Plant Stress Responses, Department of Applied Biological Science, Faculty of Agriculture, Kagawa UniversityKagawa, Japan; ^2^Department of Agronomy, Faculty of Agriculture, Sher-e-Bangla Agricultural UniversityDhaka, Bangladesh; ^3^Department of Agricultural Botany, Faculty of Agriculture, Sher-e-Bangla Agricultural UniversityDhaka, Bangladesh

**Keywords:** calcium, methylglyoxal, nutrient homeostasis, osmotic stress, oxidative stress, salinity

## Abstract

The present study investigates the regulatory role of exogenous calcium (Ca) in developing salt stress tolerance in rice seedlings. Hydroponically grown 13-day-old rice (*Oryza sativa* L. cv. BRRI dhan47) seedlings were exposed to 200 mM NaCl alone and combined with 2 mM CaCl_2_ and 2 mM ethylene glycol tetraacetic acid (EGTA, a Ca scavenger) for 3 days. The salt stress caused growth inhibition, chlorosis and water shortage in the rice seedlings. The salt-induced stress disrupted ion homeostasis through Na^+^ influx and K^+^ efflux, and decreased other mineral nutrient uptake. Salt stress caused oxidative stress in seedlings through lipid peroxidation, loss of plasma membrane integrity, higher reactive oxygen species (ROS) production and methylglyoxal (MG) formation. The salt-stressed seedlings supplemented with exogenous Ca recovered from water loss, chlorosis and growth inhibition. Calcium supplementation in the salt-stressed rice seedlings improved ion homeostasis by inhibition of Na^+^ influx and K^+^ leakage. Exogenous Ca also improved ROS and MG detoxification by improving the antioxidant defense and glyoxalase systems, respectively. On the other hand, applying EGTA along with salt and Ca again negatively affected the seedlings as EGTA negated Ca activity. It confirms that, the positive responses in salt-stressed rice seedlings to exogenous Ca were for Ca mediated improvement of ion homeostasis, antioxidant defense and glyoxalase system.

## Introduction

Gradual changes in climate challenges crop production through various abiotic stresses, which become major constraints to crop production due to the unpredictable and complex nature of the environment (Mittler and Blumwald, 2010). Of the environmental factors, salinity is one of the most devastating abiotic stresses because most crop plants are sensitive to salt stress (Hasanuzzaman et al., 2013). Around 20% of irrigated land has been affected by salinity (Pitman and Lauchli, 2002), and it is estimated that salt stress will cause up to 50% loss of cultivable land in the middle of the 21st century (Mahajan and Tuteja, 2005). Plants exposed to higher levels of salinity are affected by both hyperionic and hyperosmotic stress through accumulating Na^+^ and Cl^-^ which causes membrane damage, nutrient imbalance, enzymatic inhibition, metabolic dysfunction, photosynthesis inhibition, and hampers other major physiological and biochemical processes that ultimately leads to growth inhibition or death of the plant (Mahajan and Tuteja, 2005; Ahmad and Sharma, 2008; Munns and Tester, 2008; Hasanuzzaman et al., 2012). Higher levels of salt in plant growth medium decrease K^+^ content and increase Na^+^ uptake as Na^+^ causes K^+^ efflux and triggers K leakage from plant cells. With higher levels of NaCl, Na displaces Ca from membranes, which also increases intracellular Na. As a result, under salt-stress conditions Na content exceeds that of K, resulting in a higher Na/K ratio as well as nutrient imbalance (Cramer et al., 1985; Shabala et al., 2006; Wu and Wang, 2012). In addition, salinity disrupts the antioxidant defense system through overproduction of reactive oxygen species (ROS) including singlet oxygen (^1^O_2_), superoxide radical (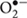), hydrogen peroxide (H_2_O_2_), and hydroxyl radicals (OH^•^), which consequently results in oxidative stress (Hasanuzzaman et al., 2013; Mishra et al., 2013). Moreover, salinity accelerates the generation of cytotoxic methylglyoxal (MG) through the glycolysis pathway and causes oxidative damage through degradation of protein synthesis (Yadav et al., 2005; Hasanuzzaman et al., 2014).

Ionic and osmotic homeostasis and ROS and MG detoxification are salt-stress tolerance mechanisms in plants (Wu and Wang, 2012; Nahar et al., 2015a). Some plants produce proline (Pro) or other compatible solutes to maintain the water relationship and stabilize protein and enzyme complexes for ionic and osmotic homeostasis (Iqbal et al., 2015; Reddy et al., 2015). On the other hand, plants normally scavenge over-produced ROS by using non-enzymatic antioxidants (ascorbic acid, AsA; glutathione, GSH; phenolic compounds; alkaloids; non-protein amino acids; and α-tocopherols) and enzymatic antioxidants (superoxide dismutase, SOD; catalase, CAT; ascorbate peroxidase, APX; glutathione reductase, GR; monodehydroascorbate reductase, MDHAR; dehydroascorbate reductase, DHAR; glutathione peroxidase, GPX; and glutathione *S*-transferase, GST; Pang and Wang, 2008; Gill and Tuteja, 2010; Hasanuzzaman et al., 2012). Detoxification of salt-induced overproduced ROS is also maintained by the antioxidant system (Anjum et al., 2014). The salt-induced higher production of MG is detoxified by the glyoxalase system, where the glyoxalase I (Gly I) and glyoxalase II (Gly II) enzymes act together with GSH (Yadav et al., 2005; Nahar et al., 2015a).

Improving ion homeostasis and regulating both the antioxidant and glyoxalase systems are necessary to develop salt-stress tolerance (Hasanuzzaman et al., 2011; Wu and Wang, 2012; Nahar et al., 2015b; Wutipraditkul et al., 2015). As an essential macronutrient, calcium (Ca) plays important roles including stabilizing cell walls and membranes, improving the metabolic processes of other nutrients, regulating enzymatic and hormonal processes, and other essential functions. Calcium also acts as a secondary messenger that mediates many aspects of cell and plant development, as well as the stress-resistance response (White and Broadley, 2003; Jaleel et al., 2007a; Jaleel and Azooz, 2009). In addition, Ca can ameliorate salt-induced Na^+^ toxicity by blocking non-selective cation channel (NSCC) which are the major pathway of Na^+^ influx in plant (Demidchik and Tester, 2002; Shabala et al., 2006). Furthermore, supplemental Ca inhibits K^+^ efflux resulted from Na^+^-induced plasma membrane depolarization (Cramer et al., 1985; Shabala et al., 2003; Tester and Davenport, 2003; Jaleel et al., 2007b; Shabala and Pottosin, 2014). Moreover, several studies have also revealed that exogenous application of Ca in plant growth medium helps to develop abiotic-stress tolerance by maintaining ion homeostasis (Wu and Wang, 2012), enhancing the antioxidant defense system and other physiological and biochemical attributes (Manivannan et al., 2007; Talukdar, 2012; Srivastava et al., 2014; Ahmad et al., 2015). Considering the above, this research was conducted to investigate maintaining the Na^+^/K^+^ ratio, and the antioxidant defense and glyoxalase systems with Ca supplementation in salt-affected rice seedlings. To the best of our knowledge, this study is the first on Ca-induced salt-stress tolerance through improving ion homeostasis and the antioxidant defense and glyoxalase systems in rice.

## Materials and Methods

### Plant Materials and Treatments

Rice (*Oryza sativa* L. cv. BRRI dhan47) seeds were surface sterilized with 70% ethanol for 8–10 min followed by washing several times with sterilized distilled water and soaked in distilled water in a dark place for 48 h. The imbibed seeds were then sown on plastic nets floating on distilled water in 250 ml plastic beakers and kept in the dark at 28 ± 2°C for 72 h. Uniformly germinated seeds were then transferred to a growth chamber (light, 350 μmol photon m^-2^ s^-1^; temperature, 25 ± 2°C; relative humidity, 65–70%) with the same pot providing a diluted (5000 times) commercial hydroponics nutrient solution (Hyponex, Japan). The nutrient solution contained 8% N, 6.43% P, 20.94% K, 11.8% Ca, 3.08% Mg, 0.07% B, 0.24% Fe, 0.03% Mn, 0.0014% Mo, 0.008% Zn, and 0.003% Cu. The nutrient solutions were renewed twice a week. Each pot contained approximately 60 seedlings. Thirteen-day-old rice seedlings were exposed to salt stress (200 mM NaCl) in presence and absence of exogenous Ca (2 mM CaCl_2_) with nutrient solution to verify the role of Ca under salt-stress conditions. We also applied the Ca scavenger ethylene glycol tetraacetic acid (C_14_H_24_N_2_O_10_; EGTA; Ammoaghaie and Moghym, 2011) together with NaCl+CaCl_2_ and alone to determine the role of Ca under salt stress conditions. Control plants were grown in Hyponex solution only. Therefore, our experiment consisted of six treatments as follows: control, 2 mM CaCl_2_ (Ca)_,_ 2 mM EGTA (EGTA), 200 mM NaCl (Salt), 200 mM NaCl+2 mM CaCl_2_ (Salt+Ca), and 200 mM NaCl+2 mM CaCl_2_+2 mM EGTA (Salt+Ca+EGTA). The experiment was repeated three times under the same conditions. Data were taken after 3 days of treatment.

### Observation of Salt Toxicity Symptoms and Seedling Growth

Seedling growth and salt toxicity symptoms in rice seedlings were determined by careful observation and measuring fresh weight (FW) and dry weight (DW). For DW, seedlings were oven dried at 70°C for 48 h. Fresh weight and DW were expressed as mg seedling^-1^. Plant height was measured from the base of shoot up to the tip of the longest leaf.

### Determination of Leaf Relative Water Content

Leaf relative water content (RWC) was measured according to Barrs and Weatherley (1962). Leaf laminas were weighed (FW), then placed immediately between two layers of filter paper and immersed in distilled water in a Petri dish for 24 h in a dark place. Turgid weight (TW) was measured after gently removing excess water with a paper towel. Dry weight was measured after 48 h oven drying at 70°C. Finally, RWC was determined using the following formula:

RWC(%) =(FW−DW)/(TW−DW)×100

### Determination of Na and Mineral Nutrient Content

Sodium, Ca, and other mineral nutrient content were determined by using an atomic absorption spectrophotometer (Hitachi Z-5000; Hitachi, Japan). Plant samples were oven dried at 70°C for 48 h. The dried samples from the roots and shoots (0.1 g) were ground and digested separately with acid mixture at 70°C for 48 h. The acid mixture consisted of HNO_3_:HClO_4_ (5:1 v/v).

### Determination of Chlorophyll Content

Chlorophyll (chl) content was measured according to Arnon (1949) by homogenizing leaf samples (0.5 g) with 10 ml of acetone (80% v/v) followed by centrifuging at 9,000×*g* for 10 min. Absorbance was measured with a UV-vis spectrophotometer at 663 and 645 nm for chl *a* and chl *b* content, respectively. Carotenoid content was also measured spectrophotometrically at wave length 470 nm.

### Determination of Proline Content

Proline content was determined according to Bates et al. (1973). Leaf samples (0.5 g) were homogenized in 5 ml 3% sulfo-salicylic acid and the homogenate was centrifuged at 11,500×*g* for 12 min. Supernatant (1 ml) was mixed with 1 ml glacial acetic acid and 1 ml acid ninhydrin. After 1 h incubation at 100°C, the mixture was cooled. The developed color was extracted with 2 ml toluene and the optical density of the chromophore was observed spectrophotometrically at 520 nm. Proline content was determined by comparing with a standard curve of known concentration of Pro.

### Histochemical Detection of ROS Markers and Membrane Damage

Localization of 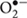 in leaves was detected following Chen et al. (2010) with a slight modification. Leaves were immersed in 0.1% Nitroblue tetrazolium chloride (NBT) solution and incubated at 25°C (temperature) for incubation. After 12 h, the incubated leaves were immersed in boiling ethanol (90%) for 15 min to decolorize the leaves and to show the dark blue spots produced by the reaction of NBT and 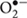.

Localization of H_2_O_2_ was detected according to the method of Thordal-Christensen et al. (1997) with a minor modification. Leaves were incubated at 25°C in a solution containing 1 mg ml^-1^ 3′,3′-diaminobenzidine (DAB) prepared in HCl acidified (pH 3.8) water. After 12 h of incubation, the leaves were boiled in ethanol (90%) for 15 min to show the reddish brown spots produced by the reaction of H_2_O_2_ and DAB.

Lipid peroxidation in the roots was determined by histochemical staining using Schiff’s reagent with a modification of Srivastava et al. (2014). Lipid peroxidation-originated aldehydes were detected after 30 min staining, indicated by pink–red color. The stained roots were rinsed with sulphite solution (0.5% [w/v] K_2_S_2_O_5_ in 0.05 M HCl) and dipped in the same solution for 10 min to retain the stained color. The loss of plasma membrane integrity in the roots was measured by histochemical staining using 0.25% aqueous Evan’s blue solution with a slight modification of Schützendübel et al. (2001). After 40 min, the roots were rinsed with distilled water and membrane damage was revealed using glycerin.

### Determination of Lipid Peroxidation and Hydrogen Peroxide Levels

The level of lipid peroxidation was measured by estimating malondialdehyde (MDA) following the method of Heath and Packer (1968). Malondialdehyde content was measured by observing the difference in absorbance at 532 nm using an extinction coefficient of 155 mM^-1^cm^-1^ and expressed as nmol of MDA g^-1^ FW. Hydrogen peroxide content was determined according to Yu et al. (2003) by observing the absorbance at 410 nm using an extinction coefficient of 0.28 μM^-1^ cm^-1^.

### Determination of Methylglyoxal Content

Methylglyoxal was measured following the method of Wild et al. (2012) by extracting plant samples in 5% perchloric acid. After centrifuging homogenized leaf tissues at 11,000×*g* for 10 min, the supernatant was decolorized by adding charcoal. The decolorized supernatant was neutralized by adding saturated sodium carbonate and used to estimate MG by adding sodium dihydrogen phosphate and *N*-acetyl-L-cysteine to a final volume of 1 ml. The absorbance was recorded after 10 min at 288 nm and MG content was calculated using a standard curve of known concentration of MG.

### Determination of Ascorbate and Glutathione Redox

Rice leaves (0.5 g) were homogenized in 3 ml ice-cold 5% meta-phosphoric acid containing 1 mM EDTA using a mortar and pestle. The homogenates were centrifuged at 11,500×*g* for 15 min at 4°C and the collected supernatants were used according to the method of Dutilleul et al. (2003) with minor modifications to determine total and reduced AsA. After neutralizing the supernatant with 0.5 M potassium-phosphate (K-P) buffer (pH 7.0), the oxidized fraction was reduced with 0.1 M dithiothreitol. Total and reduced AsA content were assayed spectrophotometrically at 265 nm in 100 mM K-P buffer (pH 7.0) with 1.0 U of ascorbate oxidase (AO). To calculate ascorbate, a specific standard curve of AsA was used. Dehydroascorbate (DHA) was measured using the formula DHA = total AsA – reduced AsA. Reduced glutathione (GSH), oxidized glutathione or glutathione disulfide (GSSG), and total glutathione (GSH + GSSG) were determined according to Griffiths (1980) based on enzymatic recycling. GSH was measured using the formula GSH = Total GSH - GSSG. Glutathione was removed by 2-vinylpyridine derivatization to determine GSSG.

### Determination of Protein

Protein concentration was measured according to Bradford (1976) using bovine serum albumin (BSA) as a protein standard.

### Enzyme Extraction and Assays

Rice leaves (0.5 g) were homogenized in 50 mM ice cold K-P buffer (pH 7.0) containing 100 mM KCl, 1 mM ascorbate, 5 mM β-mercaptoethanol, and 10% (w/v) glycerol using a pre-cooled mortar and pestle. The homogenates were centrifuged two times at 11,500×*g* for 15 min and the supernatants were used to determine protein content and enzyme activity. All procedures were performed at 0–4°C.

Lipoxygenase (LOX, EC: 1.13.11.12) activity was determined according to the method of Doderer et al. (1992) using linoleic acid as a substrate solution. The increased absorbance was observed at 234 nm and the activity was calculated using an extinction coefficient of 25 mM^-1^cm^-1^.

Ascorbate peroxidase (APX, EC: 1.11.1.11) activity was determined according to Nakano and Asada (1981) by observing the decreased absorbance at 290 nm for 1 min and using an extinction coefficient of 2.8 mM^-1^cm^-1^. The reaction buffer solution contained 50 mM K-P buffer (pH 7.0), 0.5 mM AsA, 0.1 mM H_2_O_2_, 0.1 mM EDTA, and enzyme extract.

Monodehydroascorbate reductase (MDHAR, EC: 1.6.5.4) activity was assayed by following the method of Hossain et al. (1984) using an extinction coefficient of 6.2 mM^-1^cm^-1^.

Dehydroascorbate reductase (DHAR, EC: 1.8.5.1) activity was determined according to the method of Nakano and Asada (1981) by observing the change in absorbance at 265 nm for 1 min using an extinction coefficient of 14 mM^-1^cm^-1^.

Glutathione reductase (GR, EC: 1.6.4.2) activity was determined according to the method of Foyer and Halliwell (1976) by monitoring the decreased absorbance at 340 nm and using an extinction coefficient of 6.2 mM^-1^cm^-1^. The reaction mixture solution contained 0.1 M K-P buffer (pH 7.8), 1 mM EDTA, 1 mM GSSG, 0.2 mM NADPH, and enzyme extract.

Glutathione *S-*transferase (GST, EC: 2.5.1.18) activity was measured as described by Hossain et al. (2006). The activity was calculated by observing the increased absorbance at 340 nm for 1 min and using an extinction coefficient of 9.6 mM^-1^cm^-1^. The reaction mixture contained 100 mM Tris-HCl buffer (pH 6.5), 1.5 mM GSH, and 1 mM 1-chloro-2,4-dinitrobenzene (CDNB), and enzyme solution.

Glutathione peroxidase (GPX, EC: 1.11.1.9) activity was determined according to Elia et al. (2003) by monitoring the change in absorbance at 340 nm for 1 min and using an extinction coefficient of 6.62 mM^-1^cm^-1^. The reaction mixture contained 100 mM K-P buffer (pH 7.5), 1 mM EDTA, 1 mM NaN_3_, 0.12 mM NADPH, 2 mM GSH, 1 unit GR, 0.6 mM H_2_O_2_, and enzyme solution.

Superoxide dismutase (SOD, EC: 1.15.1.1) activity was measured based on the xanthine-xanthine oxidase system following the method of El-Shabrawi et al. (2010).

Catalase (CAT, EC: 1.11.1.6) activity was measured as described by Hasanuzzaman and Fujita (2011) using an extinction coefficient of 39.4 mM^-1^cm^-1^.

Glyoxalase I (Gly I, EC: 4.4.1.5) and Glyoxalase II (Gly II, EC: 3.1.2.6) activities were determined as described by Hasanuzzaman and Fujita (2011) using extinction coefficient of 3.37 mM^-1^cm^-1^ and 13.6 mM^-1^cm^-1^, respectively.

### Statistical Analysis

The data were subjected to analysis of variance (ANOVA) and the mean differences were compared by Fisher’s LSD using XLSTAT v.2015 software (Addinsoft, 2015). Differences at *P* ≤ 0.05 were considered significant.

## Results

### Phenotypic Appearance

**FIGURE 1:**
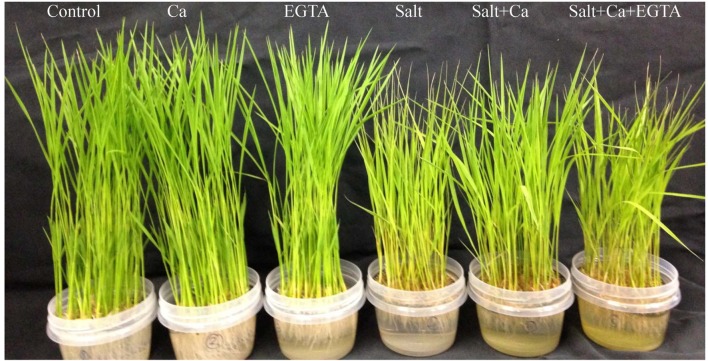
**Phenotypic appearance of rice seedlings under different treatments.** Here, Ca, EGTA and Salt indicate 2 mM CaCl_2_, 2 mM EGTA, and 200 mM NaCl, respectively.

Salt stress severely damaged the rice seedlings phenotypically, resulting in the rolling and burning of leaf tips and the yellowing of the whole plant. Supplementation with Ca partially reversed the salt-induced damage and improved the phenotypic appearance of the seedlings. The simultaneous application of Ca and EGTA damaged rice seedlings again phenotypically with the Salt+Ca+EGTA treatment, compared with the Salt+Ca treatment (**Figure 1**). However, under non-stress conditions, exogenous Ca and EGTA had no visual effect on the rice seedlings.

### Plant Growth

Salt-induced stress decreased plant growth in terms of plant height, seedling FW and DW (**Table 1**). Supplementation with Ca to the salt-treated seedlings markedly restored plant growth compared the seedlings treated with salt alone. Applying EGTA to the Salt+Ca+EGTA treatment decreased plant growth again, which indicate that EGTA, as a Ca scavenger, negates the activity of Ca which increases plant growth. However, applying Ca and EGTA without salt stress did not affect plant growth.

**Table 1:** Effect of Ca on growth parameter, leaf relative water content (RWC) and proline content in leaf of rice seedlings under salt stress.

Treatment	Plant height (cm)	Fresh weight (mg seedling^-1^)	Dry weight (mg seedling^-1^)	Leaf RWC (%)	Proline content (μmol g^-1^ FW)
Control	23.3 ± 0.32^a^	151.80 ± 9.45^a^	19.00 ± 0.46^a^	98.37 ± 0.66^a^	0.20 ± 0.01^b^
Ca	23.16 ± 0.24^a^	144.10 ± 7.36^a^	18.63 ± 0.44^a^	97.88 ± 0.25^a^	0.21 ± 0.02^b^
EGTA	23.26 ± 0.19^a^	142.77 ± 9.46^a^	18.80 ± 0.50^a^	97.57 ± 0.86^a^	0.21 ± 0.02^b^
Salt	18.86 ± 0.13^c^	98.33 ± 5.82^b^	14.87 ± 0.54^b^	79.45 ± 1.51^c^	3.73 ± 0.29^a^
Salt+Ca	20.10 ± 0.12^b^	136.33 ± 6.71^a^	18.50 ± 0.78^a^	93.08 ± 1.62^b^	0.25 ± 0.02^b^
Salt+Ca+EGTA	18.47 ± 0.32^c^	100.67 ± 8.31^b^	15.97 ± 0.99^b^	79.56 ± 1.52^c^	3.61 ± 0.17^a^


*Means (±SD) were calculated from three replicates for each treatment. Values with different letters are significantly different at *P* ≤ 0.05 applying the Fisher’s LSD test. Here, Ca, EGTA and salt indicate 2 mM CaCl_2_, 2 mM EGTA, and 200 mM NaCl, respectively.*

### Leaf RWC

Salt stress significantly reduced leaf RWC of the rice seedlings, compared with the control seedlings. Exogenous application of Ca to the salt-stressed seedlings improved RWC compared with the seedlings treated with salt alone. Adding the Ca scavenger EGTA again reduced leaf RWC compared with the Salt+Ca+EGTA treatment (**Table 1**).

### Proline Content

Treating the rice seedlings with salt considerably increased Pro content compared with the control seedlings, whereas Ca supplementation reduced Pro content compared with the seedlings treated with salt alone. However, applying Ca and EGTA without salt stress did not affect the rice seedlings (**Table 1**).

### Photosynthetic Pigments

Rice seedlings treated with salt resulted in decreased chl *a*, chl *b*, chl (*a*+*b*) and carotenoid content by 33, 38, 34, and 38%, respectively, compared with control seedlings (**Table 2**). Exogenous application of Ca to the salt-treated rice seedlings improved chl and carotenoid content compared with the seedlings treated with salt alone, but applying the Ca scavenger decreased chl and carotenoid content again in the Salt+Ca+EGTA treatment.

**Table 2:** Effect of Ca on chl and carotenoid contents in leaf of rice seedlings under salt stress.

Treatment	chl *a* (mg g^-1^ FW)	chl *b* (mg g^-1^ FW)	chl (*a*+*b*) (mg g^-1^ FW)	Carotenoid (mg g^-1^ FW)
Control	2.48 ± 0.1^a^	0.73 ± 0.03^a^	3.22 ± 0.08^a^	0.56 ± 0.02^a^
Ca	2.42 ± 0.09^a^	0.70 ± 0.04^ab^	3.11 ± 0.13^ab^	0.54 ± 0.01^a^
EGTA	2.42 ± 0.1^a^	0.73 ± 0.05^a^	3.14 ± 0.12^ab^	0.55 ± 0.02^a^
Salt	1.66 ± 0.03^b^	0.45 ± 0.02^c^	2.11 ± 0.05^c^	0.35 ± 0.02^c^
Salt+Ca	2.30 ± 0.13^a^	0.66 ± 0.02^b^	2.96 ± 0.14^b^	0.46 ± 0.02^b^
Salt+Ca+EGTA	1.71 ± 0.13^b^	0.47 ± 0.01^c^	2.18 ± 0.12^c^	0.36 ± 0.03^c^


*Means (±SD) were calculated from three replicates for each treatment. Values with different letters are significantly different at *P* ≤ 0.05 applying the Fisher’s LSD test. Here, Ca, EGTA and salt indicate 2 mM CaCl_2_, 2 mM EGTA, and 200 mM NaCl, respectively.*

### Na^+^ and K^+^ Homeostasis

**FIGURE 2:**
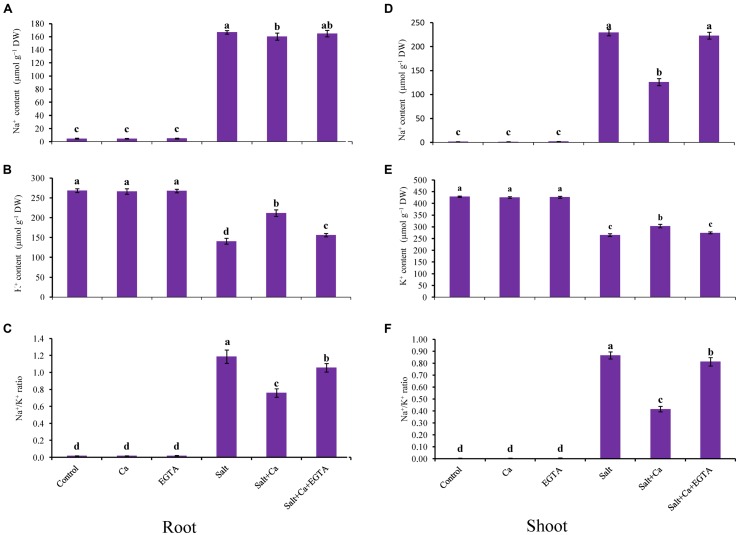
**Effect of Ca on Na^+^ and K^+^ contents and their ratio in root **(A,B,C)** and shoot **(D,E,F)** of rice seedlings under salt stress.** Here, Ca, EGTA, and Salt indicate 2 mM CaCl_2_, 2 mM EGTA, and 200 mM NaCl, respectively. Means (±SD) were calculated from three replicates for each treatment. Bars with different letters are significantly different at *P* ≤ 0.05 applying the Fisher’s LSD test.

A marked increase in Na^+^ uptake was observed in the shoots of the salt-treated rice seedlings compared with the control seedlings (**Figures 2A,D**). Exogenous Ca reduced the Na^+^ uptake in the shoots: it was 45% lower in the Salt+Ca treatment compared with the salt-alone treatment. The Ca scavenger EGTA enhanced Na^+^ uptake again in the Salt+Ca+EGTA treatment, which indicates that EGTA negates the activity of exogenous Ca. The K^+^ content in the roots and shoots decreased significantly in the salt-affected rice seedlings compared with the non-stressed control seedlings. The lower K^+^ uptake partially recovered in the Salt+Ca treatment compared with the salt-treated rice seedlings and decreased again in the Salt+Ca+EGTA treatment (**Figures 2B,E**). The ratio of Na^+^/K^+^ increased in the roots and shoots in the salt-stressed seedlings and decreased with Ca supplementation (**Figures 2C,F**). However, exogenous application of Ca and EGTA together to the salt-affected seedlings increased the Na^+^/K^+^ ratio in the Salt+Ca+EGTA treatment compared with the Salt+Ca treatment. Under non-stress conditions, Ca and EGTA did not affect on Na^+^ content, K^+^ content or their ratio in both the roots and shoots.

### Mineral (Ca, Mg, Mn, and Zn) Nutrient Contents

In the salt-treated rice seedlings, the mineral nutrient content decreased in the roots and shoots compared with the control seedlings. The stress induced by salt decreased the Ca, Mg, Mn, and Zn content in the shoots by 29, 14, 37, and 31%, respectively, compared with the control seedlings. However, Ca supplementation increased the mineral nutrient content (except for Mn) of the shoots in the Salt+Ca treatment compared with the salt-treated rice seedlings. In contrast, the mineral nutrient content in the roots and shoots decreased again with the Salt+Ca+EGTA treatment compared with the Salt+Ca treatment (**Table 3**). Adding exogenous Ca slightly decreased the Mn content in the roots and shoots under non-stress conditions. However, exogenous application of EGTA to the non-stress control seedlings did not affect on mineral nutrient content except for Ca, where EGTA decreased the Ca content in the roots and shoots.

**Table 3:** Effect of Ca on mineral nutrient contents of rice seedlings under salt stress.

Treatment	Ca content (μ mol g^-1^ DW)		Mg content (μ mol g^-1^ DW)		Mn content (μ mol g^-1^ DW)		Zn content (μ mol g^-1^ DW)	
				
	Root	Shoot	Root	Shoot	Root	Shoot	Root	Shoot
Control	39.47 ± 1.76^c^	131.94 ± 5.81^b^	69.91 ± 3.44^a^	115.17 ± 2.79^a^	0.18 ± 0.01^a^	0.19 ± 0.01^a^	0.16 ± 0.01^a^	0.19 ± 0.01^a^
Ca	57.79 ± 1.90^a^	167.51 ± 5.54^a^	70.47 ± 5.14^a^	113.16 ± 2.21^a^	0.15 ± 0.01^b^	0.17 ± 0.01^b^	0.16 ± 0.01^a^	0.19 ± 0.01^a^
EGTA	35.61 ± 2.29^d^	119.36 ± 3.21^c^	69.70 ± 1.99^a^	113.13 ± 3.32^a^	0.18 ± 0.01^a^	0.19 ± 0.01^a^	0.16 ± 0.01^a^	0.18 ± 0.01^a^
Salt	36.58 ± 1.01^cd^	93.34 ± 2.69^e^	36.37 ± 3.09^d^	99.48 ± 2.55^c^	0.13 ± 0.01^c^	0.12 ± 0.01^c^	0.11 ± 0.01^c^	0.13 ± 0.01^c^
Salt+Ca	47.27 ± 1.40^b^	111.57 ± 1.23^d^	53.94 ± 2.91^b^	108.34 ± 1.07^b^	0.14 ± 0.01^c^	0.16 ± 0.01^b^	0.14 ± 0.01^b^	0.16 ± 0.01^b^
Salt+Ca+EGTA	38.61 ± 1.71^cd^	95.56 ± 1.21^e^	43.69 ± 2.92^c^	102.6 ± 1.84^c^	0.12 ± 0.1^c^	0.12 ± 0.01^c^	0.11 ± 0.01^c^	0.13 ± 0.01^c^


*Means (±SD) were calculated from three replicates for each treatment. Values with different letters are significantly different at *P* ≤ 0.05 applying the Fisher’s LSD test. Here, Ca, EGTA and Salt indicate 2 mM CaCl_2_, 2 mM EGTA, and 200 mM NaCl, respectively.*

### ROS Generation, Lipid Peroxidation and Membrane Damage

**FIGURE 3:**
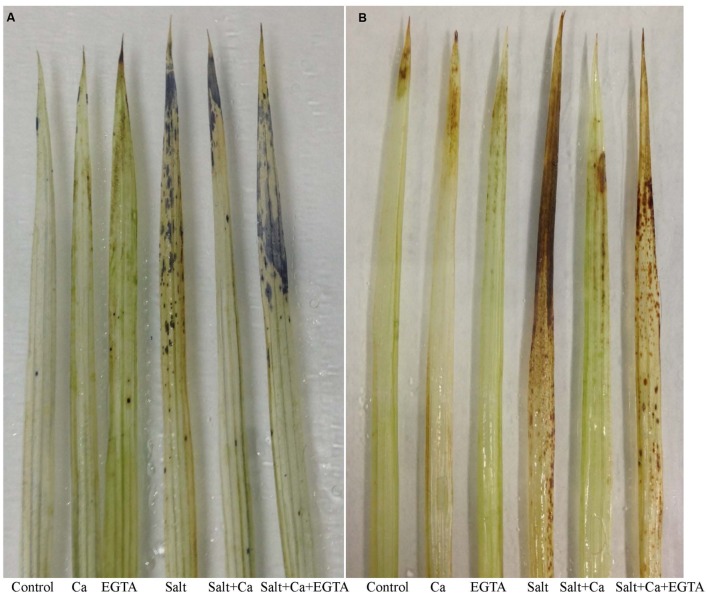
**Histochemical detection of 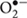
**(A)** and H_2_O_2_**(B)** in leaf of rice seedlings under salt stress.** Here, Ca, EGTA and Salt indicate 2 mM CaCl_2_, 2 mM EGTA, and 200 mM NaCl, respectively.

**FIGURE 4:**
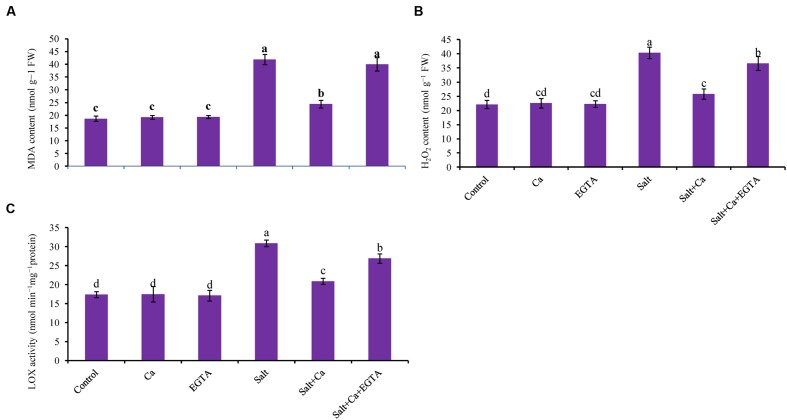
**Effect of Ca on lipid peroxidation (MDA content) **(A)**, H_2_O_2_ content **(B)** and LOX activity **(C)** in leaf of rice seedlings under salt stress.** Here, Ca, EGTA and Salt indicate 2 mM CaCl_2_, 2 mM EGTA, and 200 mM NaCl, respectively. Means (±SD) were calculated from three replicates for each treatment. Bars with different letters are significantly different at *P* ≤ 0.05 applying the Fisher’s LSD test.

The salt-induced stress caused overproduction of ROS in the rice seedlings. The increased ROS content resulted in oxidative damage to the rice seedlings leading to increased lipid peroxidation and cell membrane damage. Histochemical staining shows over-production of 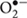 and H_2_O_2_ with dark blue spots and brown spots, respectively, in the salt-treated seedlings (**Figures 3A,B**). Applying Ca to the salt-treated seedlings considerably reduced the spots of 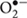 and H_2_O_2,_ compared with salt-alone treatment. Salt exposure also resulted in an increase in H_2_O_2_ level, which was 82% higher compared with the control seedlings (**Figure 4B**). Adding Ca to the salt-treated seedlings played a positive role in reducing H_2_O_2_ level. However, applying EGTA increased the production of 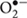 (**Figure 3A**) and H_2_O_2_ (**Figures 3B** and **4B**) again in the Salt+Ca+EGTA treatment as EGTA negated the Ca activity.

**FIGURE 5:**
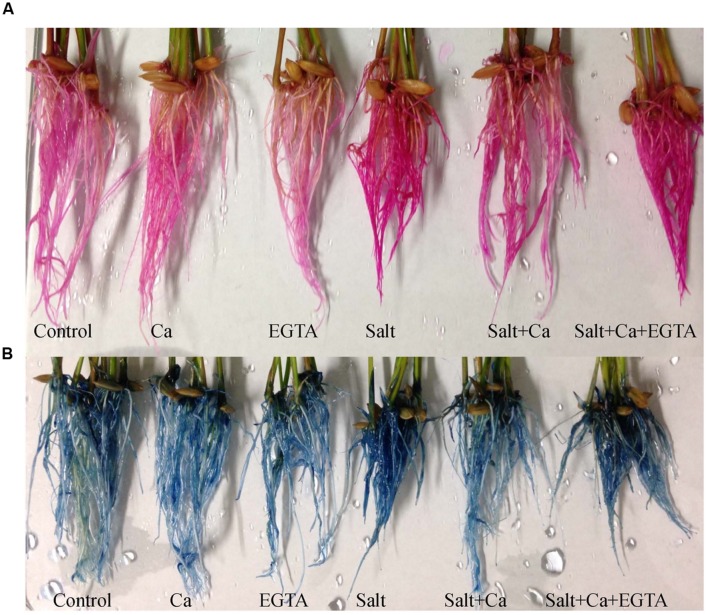
**Histochemical detection of lipid peroxidation **(A)** and loss of plasma membrane integrity **(B)** in root of rice seedlings under salt stress.** Here, Ca, EGTA and Salt indicate 2 mM CaCl_2_, 2 mM EGTA, and 200 mM NaCl, respectively.

The oxidative stress-induced lipid membrane damage was measured using lipid peroxidation in terms of MDA content in the leaves and by histochemical staining of the roots using Schiff’s reagent. Membrane damage was also identified by staining the roots with Evan’s blue dye and then observing plasma membrane integrity (**Figures 5A,B**). Compared with the control seedlings, a significant rise in MDA content was observed in the leaves of salt-treated rice seedlings (**Figure 4A**). Histochemical staining also showed higher lipid peroxidation indicated by an intense pink–red color and higher loss of plasma membrane integrity indicated by an intense dark blue color in the roots of salt-treated rice seedlings (**Figures 5A,B**). Exogenous application of Ca reduced the membrane damage, indicated by a 42% reduction in MDA content, and a reduction in the intense color formation during histochemical staining of the roots in the Salt+Ca treatment compared with the salt-treated rice seedlings. However, the Ca scavenger EGTA increased membrane damage again in the Salt+Ca+EGTA treatment compared with the Salt+Ca treatment.

### LOX Activity

Lipoxygenase activity increased by 78% in the salt-treated rice seedlings compared with the control seedlings which were decreased with Ca supplementation (**Figure 4C**). In non-stress conditions, exogenous Ca and EGTA did not affect rice seedlings compared with the control but adding EGTA increased LOX activity again in the Salt+Ca+EGTA treatment.

### Ascorbate and Glutathione Redox System

**FIGURE 6:**
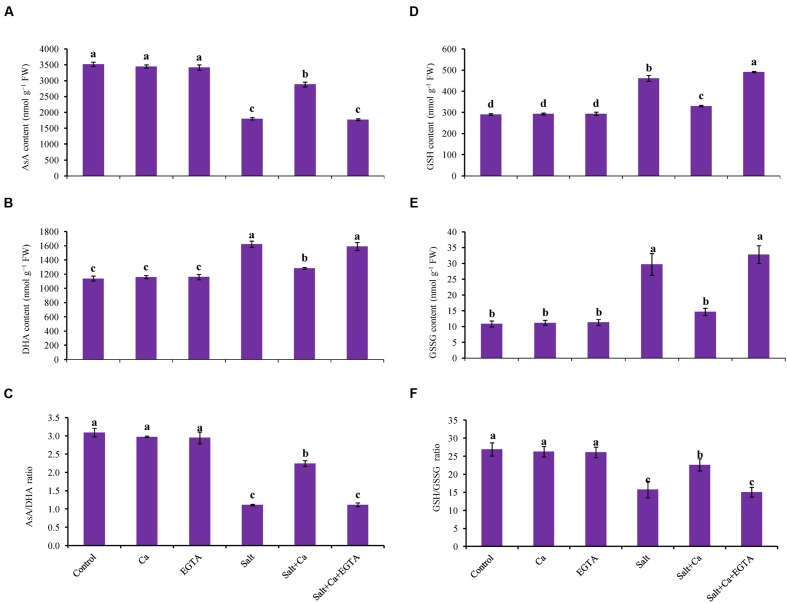
**Effect of Ca on AsA content **(A)**, DHA content **(B)**, AsA/DHA ratio **(C)**, GSH content **(D)**, GSSG content **(E)**, and GSH/GSSG ratio **(F)** in leaf of rice seedlings under salt stress.** Here, Ca, EGTA and Salt indicate 2 mM CaCl_2_, 2 mM EGTA, and 200 mM NaCl, respectively. Means (±SD) were calculated from three replicates for each treatment. Bars with different letters are significantly different at *P* ≤ 0.05 applying the Fisher’s LSD test.

Treatment of rice seedlings with salt decreased AsA content by 49%, which was partly improved with Ca supplementation in the Salt+Ca treatment (**Figure 6A**). Applying EGTA to the Salt+Ca+EGTA treatment decreased AsA content again. Dehydroascorbate content increased in the salt-treated rice seedlings and decreased with Ca supplementation (**Figure 6B**). The ratio of AsA/DHA decreased with salt stress which improved with Ca supplementation in salt-stressed rice seedlings. Applying EGTA in the Salt+Ca+EGTA treatment increased DHA content and decreased AsA/DHA ratio (**Figure 6C**). However, applying Ca and EGTA to the non-stressed control seedlings did not affect AsA and DHA content or their ratio.

Glutathione and GSSG content significantly increased in the salt-treated rice seedlings compared with the control seedlings (**Figures 6D,E**). The ratio of GSH/GSSG decreased by 42% in the salt-stressed rice seedlings (**Figure 6F**). Exogenous Ca reduced the GSH and GSSG content but increased the GSH/GSSG ratio in the Salt+Ca treatment compared with salt-stressed rice seedlings. Applying EGTA increased the GSSG content by 55% and decreased the GSH/GSSG ratio by 33% in the Salt+Ca+EGTA treatment compared with the Salt+Ca treatment. However, exogenous Ca and EGTA did not affect the non-stressed control seedlings (**Figures 6E,F**).

### Activities of Antioxidant Enzymes

**FIGURE 7:**
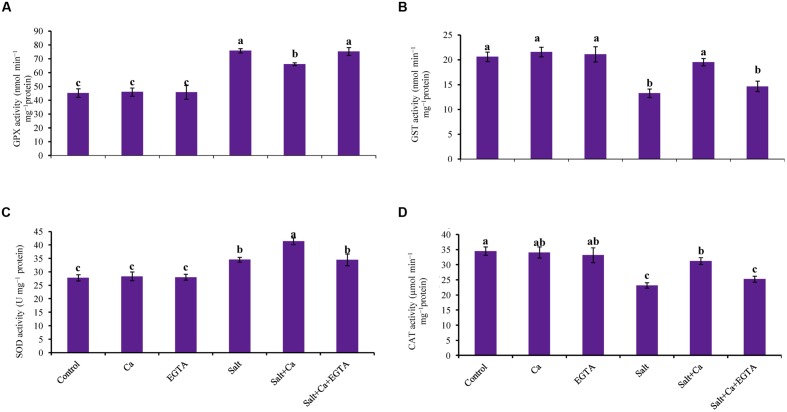
**Effect of Ca on GPX **(A)**, GST **(B)**, SOD **(C)**, and CAT **(D)** activity in leaf of rice seedlings under salt stress.** Here, Ca, EGTA and Salt indicate 2 mM CaCl_2_, 2 mM EGTA, and 200 mM NaCl, respectively. Means (±SD) were calculated from three replicates for each treatment. Bars with different letters are significantly different at *P* ≤ 0.05 applying the Fisher’s LSD test.

Superoxide dismutase activity increased by 24% in the rice seedlings subjected to salt, compared with control. Calcium supplementation further increased SOD activity by 20% in the Salt+Ca treatment compared with the salt-treated rice seedlings. Compared with the Salt+Ca treatment, applying EGTA decreased SOD activity in the Salt+Ca+EGTA treatment. However, in non-stress conditions, exogenous application of Ca and EGTA did not affect SOD activity (**Figure 7C**).

The rice seedlings, treated with salt had decreased CAT activity by 33% compared with control. In contrast, Ca supplementation increased CAT activity in the salt-treated rice seedlings by 35% compared with the seedlings treated with salt alone. Exogenous Ca and EGTA did not affect CAT activity in the non-stressed control seedlings (**Figure 7D**).

**FIGURE 8:**
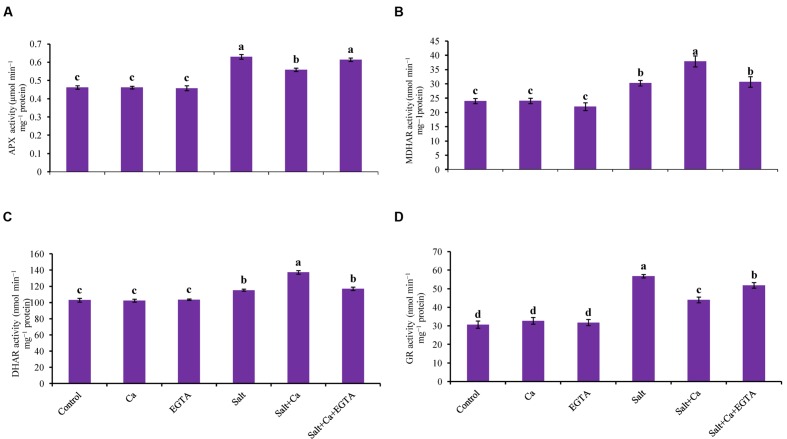
**Effect of Ca on APX **(A)**, MDHAR **(B)**, DHAR **(C)**, and GR **(D)** activity in leaf of rice seedlings under salt stress.** Here, Ca, EGTA and Salt indicate 2 mM CaCl_2_, 2 mM EGTA, and 200 mM NaCl, respectively. Means (±SD) were calculated from three replicates for each treatment. Bars with different letters are significantly different at *P* ≤ 0.05 applying the Fisher’s LSD test.

The rice seedlings exposed to salt stress had increased APX activity compared with control. Exogenous application of Ca decreased APX activity compared with salt-treated rice seedlings. However, exogenous Ca and EGTA did not affect on APX activity under non-stress conditions (**Figure 8A**).

Salt stress increased MDHAR activity in the rice seedlings compared with the control seedlings. Exogenous Ca further increased MDHAR activity by 25% in the Salt+Ca treated rice seedlings. Applying EGTA decreased MDHAR activity again in the Salt+Ca+EGTA treatment compared with the Salt+Ca treatment (**Figure 8B**).

Exogenous application of Ca increased DHAR activity in the Salt+Ca-treated rice seedlings compared with salt-treated seedlings. However, exogenous application of EGTA decreased DHAR activity by 15% in the Salt+Ca+EGTA treatment compared with the Salt+Ca treated seedlings (**Figure 8C**).

Glutathione reductase activity increased with the Salt and Salt+Ca+EGTA treatment, compared with the control seedlings. In contrast with the salt-treated rice seedlings, Ca supplementation did not increase GR activity in the Salt+Ca treated rice seedlings. Exogenous application of Ca and EGTA did not affect on GR activity in non-stress conditions (**Figure 8D**).

Compared with the control seedlings, GPX activity increased in the salt-treated seedlings by 67%. Adding Ca decreased GPX activity in Salt+Ca treatment but slightly increased together with EGTA in the Salt+Ca+EGTA treatment (**Figure 7A**).

Salt stress reduced GST activity in the salt-treated rice seedlings. Compared with the salt-treated rice seedlings, GST activity increased by 47% in the Salt+Ca treatment. Compared with the control seedlings, exogenous Ca and EGTA did not affect on GST activity in non-stress conditions (**Figure 7B**).

### Glyoxalase System

**FIGURE 9:**
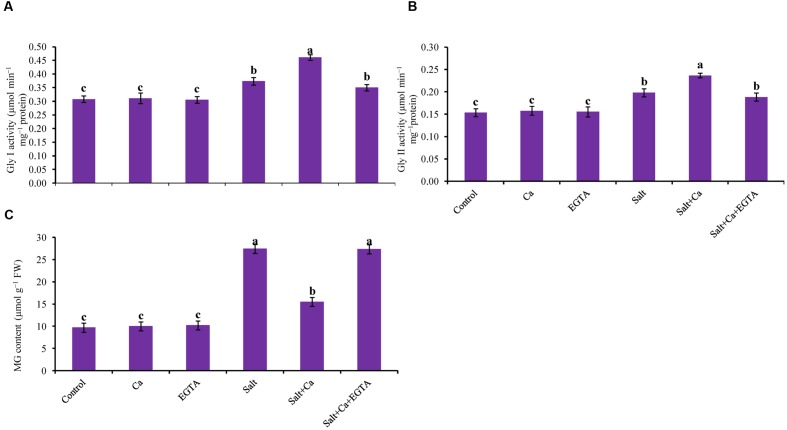
**Effect of Ca on Gly I **(A)** and Gly II activity **(B)**, and MG content **(C)** in leaf of rice seedlings under salt stress.** Here, Ca, EGTA and Salt indicate 2 mM CaCl_2_, 2 mM EGTA, and 200 mM NaCl, respectively. Means (±SD) were calculated from three replicates for each treatment. Bars with different letters are significantly different at *P* ≤ 0.05 applying the Fisher’s LSD test.

Rice seedlings exposed to salt had sharply increased MG content compared with the non-stressed control seedlings. Adding Ca to the salt-treated rice seedlings reduced MG content by 44% in the Salt+Ca treatment compared with the salt-treated rice seedlings. Applying the Ca scavenger EGTA increased MG content again in the Salt+Ca+EGTA treatment compared with the Salt+Ca treated seedlings. Adding Ca and EGTA to the non-stressed control seedlings did not result in a marked change in MG content compared with the control seedlings (**Figure 9C**).

Glyoxalase I and Gly II activity increased in the salt-treated rice seedlings by 21 and 29%, respectively, compared with the control seedlings. However, exogenous application of Ca further increased Gly I and Gly II activity by 24 and 20%, respectively, in the Salt+Ca treatment, compared with the control seedlings (**Figures 9A,B**).

## Discussion

The immediate and primary response of plants exposed to higher salt is Na^+^-induced K^+^ efflux (Nedjimi and Daoud, 2009; Anschutz et al., 2014; Bose et al., 2014). In the presence of excess salt in plant growth medium, Na^+^ influx depolarizes the root plasma membrane which activate guard cell outward rectifying potassium channels (GORK), that provide a pathway for diffusion of Na^+^ into cell and concomitantly decrease cytosolic K^+^ and increase Na^+^ content (Blumwald et al., 2000; Demidchik and Tester, 2002). Higher salt in plant growth medium also displaces Ca from membranes, which also increases membrane permeability and intracellular Na^+^ concentration (Lahaye and Epstein, 1969). Higher Na^+^ content from salt-induced stress causes disruption of the Na^+^/K^+^ ratio (Simaei et al., 2012) and ion homeostasis (Tuncturk et al., 2008). In this study, salt-induced stress increased Na^+^ content and decreased K^+^ content in the shoots and roots of rice seedlings which might be due to entry of higher amount of Na^+^ into plant by NSCC that caused K^+^ efflux or leakage through NSCC and GORK channel. Higher Na^+^ accumulation also results in a higher Na^+^/K^+^ ratio, which disrupts ion homeostasis by decreasing Mg, Mn, and Zn contents. Decreased Ca content was also observed in the salt-affected rice seedlings, which might be due to displacement of Ca by Na^+^. The Na^+^ influx and K^+^ leakage might be also for higher ROS production that can also activate NSCC (Demidchick and Maathuis, 2007). Similar disruption of ion homeostasis under salt stress conditions was demonstrated in previous studies (Tuncturk et al., 2008; Wu and Wang, 2012). However, it is reported that exogenous Ca promotes membrane stability, ameliorate salt toxicity by decreasing Na^+^ influx through NSCC and inhibiting K^+^ efflux through GORK channel in plants (Cramer et al., 1985; Essah et al., 2003; Shabala et al., 2006; Nedjimi and Daoud, 2009; Shabala and Pottosin, 2014). Moreover, exogenous Ca decreases the uptake and transport of Na^+^ and prevents binding to the cell wall (Kurth et al., 1986; Rubio et al., 2003). In addition, vacuolar and cytosolic Ca block the fast vacuole (FV) channel in voltage dependent and independent manner (Tikhonova et al., 1997) which prevent back leaking of Na^+^ into vacuole and their transportation into the cell (Shabala, 2013). In our study, exogenous application of Ca improved ion homeostasis by decreasing Na^+^ uptake, ROS production and increasing mineral nutrient uptake including K^+^ and Ca. The reduction of Na^+^ content and improvement of K^+^ retention might be due to Ca-induced block in NSCC and GORK channel. The reduction of Na^+^ accumulation was higher in shoot compared with root might be due to Ca-induced block in FV channel which inhibit further transport of Na^+^ from root to shoot. These results are in agreement with Wu and Wang (2012), who reported that exogenous Ca regulates K^+^/Na^+^ homeostasis by decreasing Na^+^ uptake and increasing K^+^ and Ca uptake. On the other hand, exogenous Ca along with EGTA could not maintain ion homeostasis in salt-stressed rice seedlings because EGTA negated Ca activity.

Under salt stress conditions higher accumulation of Na^+^ disrupts ion homeostasis, causes osmotic stress and inhibits growth (Tuncturk et al., 2008; Munns, 2011). Salt-affected rice seedlings showed growth inhibition in terms of plant height, seedling FW and DW, which were restored with Ca supplementation. This mitigation of growth inhibition under salt stress might be due to the improved ion homeostasis with Ca supplementation. This result is consistent with previous findings (Manivannan et al., 2007; Rahman et al., 2015b) in which exogenous Ca restored growth under abiotic-stress conditions.

Since salt stress causes both ionic toxicity and osmotic stress which creates physiological drought due to exosmosis and interruption of water uptake (Munns, 2011). As an osmoprotectant with antioxidant potential, Pro plays a vital role in abiotic-stress tolerance in plants (Hasanuzzaman et al., 2014; Nahar et al., 2016). Rice seedlings exposed to salt showed lower RWC and higher Pro accumulation, which indicated a salt-induced water imbalance and osmotic stress. Similar salt-induced water shortage and Pro accumulation were observed in salt-affected rice seedlings (Hasanuzzaman et al., 2014). However, exogenous Ca restored water loss (indicated by increased RWC) and decreased Pro accumulation in the salt-affected rice seedlings.

Like other abiotic stresses, salt-induced stress destabilizes the pigment protein complex and decreases photosynthetic pigments by increasing the activity of chlorophyllase enzyme and/or overproduction of ROS (Saha et al., 2010; Hasanuzzaman et al., 2014). In our experiment, we observed that Ca supplementation restored chl and carotenoid content of the rice seedlings under salt-stress conditions. The restoration of photosynthetic pigment might be due to lower production of ROS with Ca supplementation under salt-stress conditions. This result is in agreement with the findings of previous studies in which Ca supplementation improved chl and carotenoid content under abiotic-stress conditions (Ahmad et al., 2015; Rahman et al., 2015b).

One of the major effects of salt stress is the production of excess amount ROS and higher lipid peroxidation (Zhu et al., 2004; Hasanuzzaman et al., 2014; Nahar et al., 2015a). Overproduction of ROS under stress causes lipid peroxidation, protein oxidation, enzyme inhibition and eventually leads to cell death (Gill and Tuteja, 2010). Oxidative stress resulting from higher ROS production increases LOX activity and causes lipid peroxidation (Molassiotis et al., 2006). Rice seedlings exposed to salt caused oxidative stress through higher ROS production, lipid peroxidation and LOX activity. However, exogenous Ca applied to the salt-stressed rice seedlings decreased oxidative stress by lowering ROS production, lipid peroxidation and LOX activity, which might be due to Ca-mediated response that is disrupted under salt stress by decreasing Ca uptake. This result is supported by previous studies (Cramer et al., 1985; Nedjimi and Daoud, 2009; Ahmad et al., 2015) in which exogenous Ca re-stabilized Ca-mediated signaling, maintained nutrient homeostasis and reduced oxidative stress. However, exogenous Ca along with EGTA could not reduce ROS production, lipid peroxidation and oxidative stress in the salt-stress induced rice seedlings because EGTA negated Ca activity. This result is in agreement with Ammoaghaie and Moghym (2011) who showed that, EGTA increased stress injury including lipid peroxidation by chelating Ca and expelling its activity.

Non-enzymatic antioxidants, (AsA and GSH), play vital a role in maintaining cellular redox potential for abiotic stress-tolerance by scavenging overproduced ROS (Pang and Wang, 2008; Mahmood et al., 2010). The primary antioxidant, AsA, directly quenches ROS by reacting with it (Gill and Tuteja, 2010) and GSH also plays an important role in scavenging ROS or toxic compounds with the help of the antioxidant enzymes GPX and GST (Szalai et al., 2009). In this study, lower AsA content and AsA/DHA ratio resulting from increased DHA content observed under salt-stress which were facilitated by higher ROS generation. However, exogenous application of Ca increased the AsA content and AsA/DHA ratio and decreased the DHA content with increased MDHAR and DHAR activity. This result is supported by Srivastava et al. (2014), who reported that exogenous Ca restored AsA content under abiotic-stress conditions by stimulating MDHAR activity. Treatment of the rice seedlings with salt increased GSH content with increased GR and decreased GST activity. The level of GSSG also increased due to oxidation of GSH to GSSG during the scavenging of ROS.

The enzymes of AsA-GSH cycle (APX, MDHAR, DHAR, and GR), together with AsA and GSH work against oxidative stress by reducing ROS production and recycling of AsA and GSH (Asada, 1992; Mishra et al., 2013). Ascorbate peroxidase mobilizes H_2_O_2_ to H_2_O via oxidation of AsA to DHA. Monodehydroascorbate reductase and DHAR regenerate AsA from DHA using NADPH and GSH as electron donors (Mishra et al., 2013). The enzyme GR together with MDHAR and DHAR also regenerates the antioxidant components such as AsA and GSH to maintain redox cellular balance (Srivastava et al., 2014). In the present study, salt-induced stress increased APX, MDHAR, DHAR, and GR activity along with decreased AsA content and increased GSH content. Stimulation of these antioxidant enzyme activities indicates that they are associated with oxidative stress tolerance (Mishra et al., 2013). The increased APX activity might be due to higher H_2_O_2_ content and lower AsA content and increased GR activity due to an increased level of GSH content under salt-stress conditions. This result is consistent with previous findings (Mishra et al., 2013; Hasanuzzaman et al., 2014; Ozfidan-Konakci et al., 2015). However, exogenous Ca applied to the salt-treated rice seedlings further stimulated MDHAR and DHAR activity along with increased AsA content and decreased DHA content. This increased MDHAR and DHAR activities might detoxify H_2_O_2_ by regenerating AsA from DHA. This result is also consistent with previous studies (Talukdar, 2012; Srivastava et al., 2014) in which it was reported that exogenous Ca stimulates MDHAR and DHAR activity under abiotic-stress condition.

Superoxide dismutase is considered a first-line enzymatic defense in controlling ROS, which converts 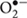 to H_2_O_2_ (Mittler, 2002; Gill et al., 2015), and H_2_O_2_ is readily detoxified to H_2_O and O_2_ by CAT (Sanchez-Casas and Klesseg, 1994). The present study showed a significant increase in SOD activity and a decrease in CAT activity under salt-stress conditions which are in agreement with previous reports (Mishra et al., 2013; Wutipraditkul et al., 2015). This increased SOD activity might be due to higher formation of 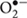 and H_2_O_2_ and decreased CAT activity due to higher production of H_2_O_2_, which were increased by salt-induced oxidative stress and supported by previous studies (Mishra et al., 2013; Hasanuzzaman et al., 2014; Nahar et al., 2015a). Exogenous application of Ca stimulated SOD and CAT activity under salt stress and reduced overproduction of 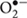 and H_2_O_2_. Similar results were reported by Srivastava et al. (2014) and Ahmad et al. (2015) who found that exogenous Ca stimulated SOD and CAT activity to reduce ROS under oxidative-stress conditions.

To protect plants from oxidative stress, the enzyme GPX and GST work together to produce less toxic and water-soluble conjugates by catalyzing the binding of different xenobiotics and their electrophilic metabolites (Edwards et al., 2000; Noctor et al., 2002). The salt-induced oxidative stress increased GPX activity but decreased GST activity might be due to higher production of H_2_O_2_ and insufficient detoxification of over-produced H_2_O_2_. This result is supported by Tammam et al. (2011) and Hasanuzzaman et al. (2014), who reported similar GPX and GST activity under salt-induced oxidative stress conditions. Supplementation with Ca in the salt-stressed rice seedlings increased GST activity, which might be playing a role in detoxifying H_2_O_2_. Similar findings were also reported by Rahman et al. (2015b), who showed stimulation of GST activity with Ca supplementation played a role in alleviating oxidative stress by scavenging H_2_O_2_.

Upregulation of the MG detoxification system or glyoxalase system is vital to eliminate overproduced MG, or MG-induced oxidative stress (Yadav et al., 2005). Overexpression of the Gly I and Gly II enzymes increased the tolerance in plants to abiotic stresses (Singla-Pareek et al., 2008). The salt-stressed rice seedlings showed higher MG production along with slight stimulation of Gly I and Gly II activity which is consistent with the findings of Hasanuzzaman et al. (2014). Supplementation of Ca in salt-treated rice seedlings showed decreased MG production with increased Gly I and Gly II activities. Decreased production of MG is partly due to increased Gly I and Gly II activities. This result is consistent with Nahar et al. (2015b), who showed that upregulation of glyoxalase enzyme activity partially resulted in oxidative-stress tolerance by increasing MG detoxification. This result is also supported by Rahman et al., 2015a,b), who showed that exogenous Ca increased MG detoxification by stimulating glyoxalase enzyme activity under toxic metal-induced oxidative stress.

## Conclusion

Considering the above, our results suggest that salinity causes disruption of ion homeostasis, and the antioxidant defense and glyoxalase systems by increasing Na^+^ uptake, ROS production and MG formation, respectively. Exogenous Ca in the salt-treated rice seedlings improved ion homeostasis by decreasing Na^+^ influx and K^+^ efflux through NSCC and GORK channel, and increasing nutrient uptake. Exogenous Ca also increased detoxification of overproduced ROS and MG by improving the antioxidant defense and glyoxalase systems under salt stress-conditions. Finally, the rice seedlings with salt-induced oxidative damage recovered with Ca supplementation, which improved ion homeostasis, and the antioxidant defense and glyoxalase systems.

## Author Contributions

AR conceived, designed, and performed the experiment and prepared the manuscript. KN actively participated in executing the experiment. MH designed the experiment and analyzed the data. MF conceived, designed, and monitored the experiment.

## Conflict of Interest Statement

The authors declare that the research was conducted in the absence of any commercial or financial relationships that could be construed as a potential conflict of interest.
